# Effects of a Theory-Based Education Program to Prevent Overweightness in Primary School Children

**DOI:** 10.3390/nu8010012

**Published:** 2016-01-04

**Authors:** Paul L. Kocken, Anne-Marie Scholten, Ellen Westhoff, Brenda P. H. De Kok, Elisabeth M. Taal, R. Alexandra Goldbohm

**Affiliations:** 1 Netherlands organization for applied scientific research (TNO), Division of Child Health, P.O. Box 3005, 2301 DA Leiden, The Netherlands; a.m.scholten@hhs.nl (A.-M.S.); Ellen.Westhoff@radboudumc.nl (E.W.); brenda.dekok@gmail.com (B.P.H.D.K.); emtaal@casema.nl (E.M.T.); 2 Leiden University Medical Center (LUMC), Department of Public Health and Primary Care, P.O. Box 9600, 2300 RC Leiden, The Netherlands; 3 The Hague University of Applied Sciences, Nutrition and Dietetics, P.O. Box 13336, 2501 EH The Hague, The Netherlands; 4 Radboud Institute for Health Sciences, Radboud university medical center, P.O. Box 9101, 6500 HB Nijmegen, The Netherlands; 5 Netherlands organization for applied scientific research (TNO), Division of Life Style, P.O. Box 3005, 2301 DA Leiden, The Netherlands; sandra.bausch@tno.nl

**Keywords:** overweight, primary school children, education program, RCT, nutrition, physical activity, behavioral determinants

## Abstract

The effectiveness of the “Extra Fit!” (EF!) education program in promoting healthy diet and physical activity to prevent and reduce overweightness among primary school children aged 9 to 11 was evaluated. A randomized controlled design was carried out in 45 primary schools (*n* = 1112) in the Netherlands, 23 intervention and 22 control schools. The intervention schools received the education program for two successive school years in grades (U.S. system) 4, 5, and 6 (mean 7.6 h during 16 weeks per school per year). The control schools followed their usual curriculum. No positive effects of EF! were found with regard to behavior and anthropometric measures when follow-up measurements were compared to the baseline. However, from baseline to follow-up after one and two school years, the intervention group improved their knowledge score significantly compared to the control group. Moreover, an effect was observed for mean time spent inactively that increased more in the control group than in the intervention group. In conclusion, limited intervention effects were found for the intervention on knowledge and inactivity. To improve the effectiveness of education programs, we advise focusing on parental involvement, attractive lessons to enlarge the acceptability of the program, and multi-component environmental strategies.

## 1. Introduction

Overweightness and obesity have become a worldwide problem [1]. Evidence from the literature shows that it is important to learn and establish healthy habits during the pre-adolescent period to prevent excessive weight gain [2,3,4]. Therefore, primary schools are a setting for the prevention of overweightness in children.

Many reviews have summarized the effectiveness of interventions to prevent overweightness in children on anthropometric measurements, behavior (food consumption, physical activity or inactivity), or behavioral determinants. The effects of such intervention studies on anthropometric measures were often absent or produced inconclusive evidence [2,5]. However, a meta-analysis by Sobol-Goldberg *et al.* [6] concluded that more recent studies showed convincing evidence that school-based programs are at least mildly effective in reducing BMI, possibly because these programs tend to be longer, more comprehensive and included parental involvement.

Behaviors most often linked to overweightness are low fruit and vegetable consumption, skipping breakfast, high fat and energy content and low fiber content of the diet and high soft drink consumption [7,8,9]. Many school-based interventions are aimed at improving fruit and vegetable consumption and small beneficial effects of such programs were reported [2]. In recent trials, Fairclough *et al.* [10] and Kipping *et al.* [11] did not find an increase in fruit and vegetable consumption, but Nyberg *et al.* [12] found a significantly higher vegetable intake in the intervention group.

Effects on physical activity turned out to be small [10], absent [11] or only present in subgroups [12]. A systematic review by Dobbins *et al.* [13] concluded that school-based physical activity interventions led to an improvement in the proportion of children who engaged in moderate to vigorous physical activity during school hours. Interventions aimed at reducing sedentary behavior (e.g., television viewing, playing games) seem to be relatively efficient both as single-component intervention and in combination with other behaviors [14]. A meta–analysis by van Grieken *et al.* [15] showed that single and multiple health behavior interventions significantly decreased sedentary behavior and BMI.

In general, although some studies reported promising effects, findings remain mixed [16] and there is doubt if beneficial results could be sustained or if they were biased by self-report. Effective interventions to prevent overweightness in primary school children have to address multiple behaviors, including nutrition, exercise and inactivity [2,7]. Moreover, to be effective, it is advised to have a theory-based foundation and address related psychosocial determinants of behavior such as attitudes, social norms and perceived behavioral control from the Theory of Planned Behavior (TPB) [7,17,18].

The study presented in this article is an evaluation of the intervention named “Extra Fit!” (EF!). The intervention combined elements of existing interventions aiming at the promotion of healthy nutrition and physical activity and the reduction of sedentary behavior and was thoroughly grounded in behavior change theory. The evaluation study used one-day food records with recall interviews and accelerometer instruments additional to self-report measurements in a two-year timeframe. The aim of EF! was to improve dietary habits, physical activity and inactivity behavior in order to prevent overweightness. The following research questions were addressed: What are the effects of the school-based program on dietary intake, physical activity and inactivity, BMI (prevalence of overweightness/obesity), and waist and hip circumference of primary school children? What are the effects of the program on related behavioral determinants: knowledge, attitude, perceived social norm, perceived behavioral control, and intention?

## 2. Experimental Section

### 2.1. Intervention

The general target of EF! was to prevent or reduce overweightness in primary school children. The Intervention Mapping (IM) procedure was used to give the program a theory base and to specify performance and change objectives aimed at the children’s dietary intake, physical activity and inactivity [19]. The methods and strategies of the classroom and physical education activities of the EF! program were based on these performance and change objectives, the TPB determinants, materials of existing Dutch school-based interventions such as “Taste Lessons” [20] that had been proven partially effective, and the framework of the effective US intervention programs “Planet Health” and “Eat Well and Keep Moving” [21,22]. The carefully developed theoretical basis and the integral approach of the dietary, physical activity and inactivity behaviors was new compared to interventions that existed at the start of EF!. EF! was developed by three Dutch national health promotion organizations, *i.e.* Netherlands Nutrition Centre, National Institute for Health Promotion (NIGZ) and Netherlands Institute for Sport and Physical Activity (NISB). Several teachers pre-tested one or more draft versions of the lessons in the school class.

The intervention EF! comprised a variety of theory and practical lessons on nutrition and physical activity to provide an attractive program for the children. The intervention was focused on the main behavioral changes: decreasing consumption of high-energy or high-fat foods and sugar-sweetened drinks; promoting a healthy breakfast; increasing consumption of fruits and vegetables; reducing television viewing and computer gaming/browsing; and increasing physical activities at school and outside school hours. The behavioral determinants of the TPB that were targeted were: knowledge (theory lessons and practical assignments), attitude (group discussions, food diaries), social norm (group discussions and homework assignments) and perceived behavioral control (modeling through assignments e.g., preparing a healthy meal and physical activity games).

EF! was targeted at 9–11 years old children and the whole school class, no distinction was made between non-overweight, overweight or obese children. The reasons for limitation of the target group to 9–11 years olds were limited budgets and the ability of primary school children of this age category to participate in paper and pencil questionnaires, used in this research. The lessons had been implemented in the regular school program during two successive school years in grades (U.S. system) 4, 5, and 6. The program consisted of seven lessons in the first school year and nine in the second year (Table S1 in Supplementary Materials). Methods that were used included theory lessons, practical lessons, homework assignments, and involvement of parents in homework. Besides, there were two optional elements: (1) an “Extra Fit-day” with appealing activities involving parents; and (2) physical activity lessons. Examples of themes that were addressed were physical activity, computer use, nutrition, and energy balance by using experiments, assignments, videos and classroom discussions. These activities were especially designed to increase knowledge and awareness of the children, to get them involved and excited, and to involve parents and teachers in the process.

### 2.2. Implementation

The EF! lessons were given by the school teachers. Collaborating local community health services and a sports service were trained to implement EF! and to give support to the teachers. The local services organized a meeting per school year in which the intervention materials were presented and discussed with the teachers in their region. The three national health promotion organizations supervised the implementation process by serving as a helpdesk for the local health and sports services and by monitoring the progress. Analysis of monitoring reports returned by the teachers of the intervention schools showed that the first lessons of the EF! program were delivered by most teachers, however there was a decline and only one out of four teachers delivered all lessons. The average total duration of the EF! lessons per year was 7.6 h (SD = 2.8) during 16 weeks per school in the intervention group. In the control schools, total time spent on education about healthy nutrition and physical activity per year was on average 3.3 h (SD = 3.9). When looking at the fidelity with respect to the involvement of parents in the intervention, it turned out that most children completed their homework without parents.

The teachers of the intervention group were moderately positive about the EF! program (score 6.7 on a scale of 1–10). Compared to the lessons of the first school year, the second year lessons were less enjoyed by the children, 17% and 39% of the children, respectively, reported not to have enjoyed most EF! lessons. The children did not find the lessons difficult and liked the physical activity lessons and the practical lessons most.

### 2.3. Design

A school-based, cluster randomized controlled study design was used to evaluate the effectiveness of EF!. The study was conducted from November 2009 to December 2011. The schools of the intervention group received the EF! program for two school years and the control schools followed their regular school program. As much as possible, matched pairs of schools were formed with similar socio-economic status (SES based on the national registry on neighborhood deprivation scores), educational level and urbanization of the region and randomized. If no similar pairs could be formed, matching at group level was sought. Informed consent procedures were followed for all children. The study was approved by the Medical Ethics Committee of Leiden University Medical Center (LUMC).

### 2.4. Participants

**Figure 1:**
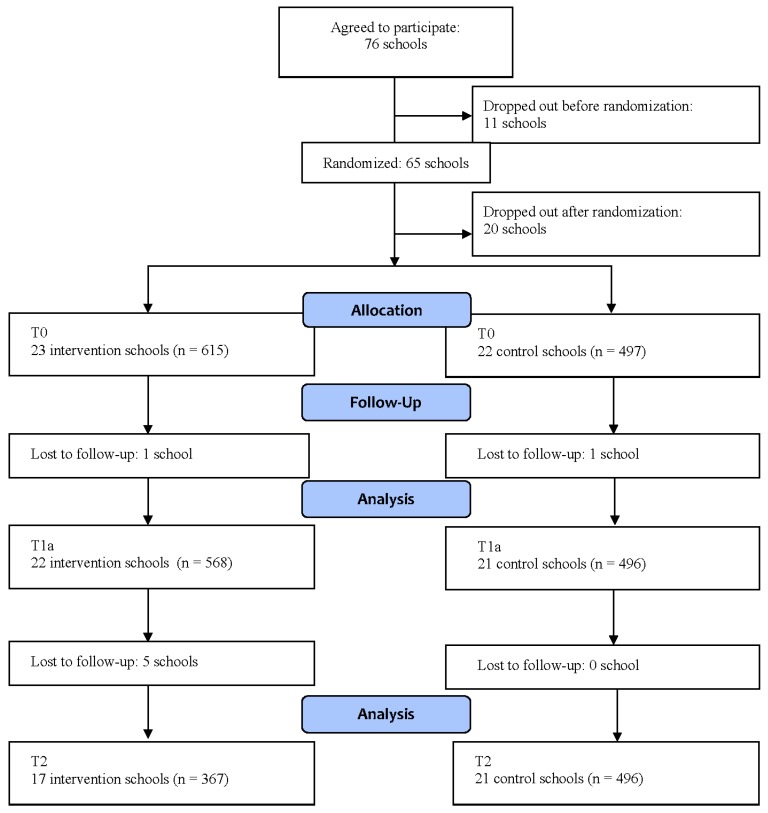
Flow diagram of schools and participants.

A total of about 500 schools were approached for participation in the study, of which 76 agreed to participate (see Figure 1). Eleven schools dropped out before the randomization procedure. The remaining schools were assigned randomly to the intervention or control condition. Before the research started, 20 schools dropped out, resulting in 23 schools in the intervention group and 22 in the control group at baseline. Reasons for schools declining were changes in personnel, the intensity of the project, or unwilling to sign a written agreement stating that the school would adhere to the research and intervention protocol. At the final measurements, there were 17 and 21 schools left, respectively. The reasons for school dropout after the study had started were change of teacher from grade 4 to 5 and lack of time.

### 2.5. Assessments

The primary outcome measures were nutrition, physical activity, sedentary behavior (inactivity and screen time) and behavioral determinants. Behavior was chosen as a primary outcome measure because the intervention was primarily aimed at behavioral change in a population with a majority of non-overweight children. BMI (prevalence of overweightness/obesity) and waist and hip circumference were secondary outcome measures. The children were assessed on four occasions during the study. The baseline measurement took place from November 2009 to December 2009 (T0). A school could start the intervention only after the baseline measurement had taken place. An intermediate follow-up took place twice, at the end of the first school year in which the children received EF! (T1a; May–July 2010) and at the end of the second school year, when the intervention schools finished the EF! program (T1b; May–July 2011). The measurement at T1b was limited and included only self-report measurement of behaviors and their determinants. The last follow-up measurement took place six months later in November–December 2011 (T2). The questionnaire was administered on all four occasions in the total study population. The anthropometric measurements were conducted three times, at T0, T1a and T2. Dietary intake and physical activity measurements using one-day records and accelerometers took place at T0 and T2, in random subsamples of the total study population. Since complete measurements would be too expensive, subgroups of approximately five to six children per school class participated in detailed measurements of dietary intake and physical activity each time. The selection procedure of the subsamples was performed in a structured manner. Parents of 10 randomly selected children per school class received a letter to gain informed consent to participate in additional measurements to get more information about the children’s dietary intake and physical activity. If parents did not respond within two weeks, they received a reminder. The selection procedure stopped when there were five to six participants per school class. This procedure was repeated at T2 and again a random sample was recruited. This resulted in cross-sectional measurements in two independent subsamples at T0 and T2. The required number of five to six children per class each time was based on sample size calculations for multilevel analysis using online (RIVM) dietary data from the Dutch National Food Consumption Survey. An overview is provided in Table 1.

**Table 1:** Overview of assessments and time points of the intervention.

Assessment	Whom	T0	T1a	T1b	T2
		0 Month	6 Months	18 Months	24 Months
Questionnaire	All children from each class	x	x	x	x
Anthropometry	All children from each class	x	x		x
Dietary record	Random sample from each class	x			x
Accelerometer	Random sample from each class	x			x
Intervention		Start after T0			

#### 2.5.1. Dietary Intake

Dietary intake was assessed twice in random subsamples consisting of 248 children with a one-day food diary at T0 and 224 children at T2. The children and parents received a food diary at home with instructions and an example food diary. Parents were instructed to help the child fill out the food diary when this was needed. To make the food diary as accurate and complete as possible, the day after the child filled out the food diary, an interview was conducted with the child together with a parent or caregiver by field workers during a home visit. The field workers checked the diary, probed for possibly forgotten items and, if the diary was very incomplete, they conducted a 24-h recall. Field workers were student dieticians, specifically trained for this study.

The data of the food diaries were systematically structured according to the Dutch food-based dietary guidelines [23] and all food items were coded based on the food items of the Dutch Food Composition Database [24]. The amount consumed per food was recorded in household measures or standard or natural units and converted into grams using a Dutch manual [25]. Furthermore, time and place of each food consumption were registered. The interviewers were blinded with respect to group status of the child’s school (intervention or control) and were equally divided between the control and intervention schools. Training and supervision of the interviewers and plausibility checks of the food diaries were conducted by an experienced dietician.

#### 2.5.2. Physical Activity

Measurement of physical activity succeeded in 122 children at T0 and 140 at T2 using a one-dimensional accelerometer; the ActiGraph. The ActiGraph measures frequency, duration and intensity of physical activity based on counts per minute, *i.e.* number of conversions of movements into electrical signals. In this study, counts per minute were collected every 15 s. In this way, also short durations of high activity were registered, which are very common in children. Parents were instructed to attach the ActiGraph to their child’s right hip by an elastic waist belt. Children should wear the ActiGraph during at least three days and remove the ActiGraph when water was involved and during sleeping time. The ActiGraph measurements were not always successful due to improper use by the child and technical problems.

#### 2.5.3. Anthropometric Measurements

Weight, height, and waist circumference measurements were completed with children in a private room at school by two trained fieldworkers. Weight was measured without shoes in clothing to the nearest 0.1 kg using a portable digital scale (SECA). Height was measured without shoes, to the nearest 0.1 cm using a portable stadiometer (SECA). Waist and hip circumference were measured to the nearest 1 mm with a flexible tape. The waist circumference was measured midway between the lower rib and the top of the iliac crest. The hip circumference was measured at the maximal circumference over the buttocks. BMI SD scores were used to categorize children as underweight, normal weight, overweight, or obese using international gender and age specific thresholds [26].

#### 2.5.4. Questionnaire

All children of each participating school class were invited to fill out questionnaires in the classroom during school-time under guidance of the teachers comparable to a normal school test. The questionnaires assessed their general characteristics (gender, age, country of birth of the child and parents), three categories of behavior (physical activity, inactivity, and dietary behavior) and six behavioral determinants derived from the TPB (knowledge, affective and cognitive attitudes, perceived social norm, perceived behavioral control and intention).

Health behavior was measured using standardized questionnaires developed by the Dutch local and national health monitor [27]. Children were asked how many days per week they were having breakfast, fruit, vegetables, sweet drinks, savory and sweet snacks and how many portions they consumed on average in a day. We studied the effect on daily breakfast or daily consumption per food item. For analysis, only the “How many days a week do you eat…?” question was taken into account due to skewness of the variables. Physical activity included questions on days per week and time per day spent on sports, outdoor play and walking or cycling to and from school. These were combined into a dichotomous outcome measure representing the proportion of children that were physically active at least 60 min each day of the week. This cut-off corresponds to the Dutch Healthy Exercise Norm (<18 years) which is at least one hour of moderately intensive physical activity (5 MET (e.g., aerobics or skateboarding) to 8 MET (e.g., running 8 km/h) every day (summer and winter), where at least twice a week the activity is aimed at improving or maintaining physical fitness (power, agility and coordination). Inactivity was assessed by combining days per week and time per day spent on watching TV and in front of a computer, resulting in three categories 1 active (0–209 min/week), 2 moderately active (210–840 min/week), and 3 inactive (>840 min/week). The Dutch recommendation for screen time for children is maximum 2 h a day, corresponding to 840 min per week.

The questions on behavioral determinants of lifestyle behaviors were adopted from other questionnaires developed in the context of overweightness and obesity prevention in the Netherlands, such as scales based on determinants of behavior change taken from the Theory of Planned Behavior (Table S2 in Supplementary Materials) [28]. An example of an item on affective attitude toward behavior is “I find a breakfast tasteful”. We also asked the children their cognitive attitude toward the frequency of behavior such as: “Do you think that you have breakfast often enough?” Knowledge of the children was measured by multiple-choice questions about the Dutch Food Pyramid (Dutch: *Schijf van Vijf*), breakfast, healthy snacks, fruit and vegetables norm, physical activity norm, types of physical activity and maximum screen time. These questions correspond to the topics that were discussed during the lessons.

### 2.6. Statistical Analysis

Energy intake, macronutrients and contribution of the food groups were calculated with the Dutch Food Composition Database [24] using the SAS application VEVES (Voeding Enquêtes Verwerkings Systeem). Food diaries that were classified as unreliable by the interviewer were excluded from the analyses. All accelerometer data were processed using the Mahuffe 1.9.0.3 software program. The data were included in the analyses if the accelerometer was worn for at least 500 min per day. Periods of zero activity counts (≥10 min) and sleeping periods were excluded. Data were expressed as mean counts per minute and as mean time spent on different intensity activities using age-specific cut off points [29,30].

Factor analysis was performed and Cronbach’s alphas were calculated to determine the behavioral determinants scales. Factor analysis showed that on the whole the behavioral determinants of specific behaviors loaded on separate factors. Therefore for each behavior a composite behavioral scale was constructed by summing up the scores of all determinant scales except knowledge into an overall score. This was done to limit multiple testing, given the many behavioral determinant variables. The Cronbach’s alphas of the composite scales varied from 0.72 to 0.77 except for sedentary behavior (screen time) that had a relatively low Cronbach’s alpha of 0.41. For dietary behavior and physical activity a score above the fiftieth percentile was considered a positive score on the composite scale. The sum score of the determinants of sedentary behavior was the only variable with a normal distribution. For this variable we used a continuous composite score.

Because schools were the sampling unit, outcome analyses were conducted using multilevel regression models, with schools included as a random effect. The model used all of the available information (*i.e.*, information from participants with both complete and incomplete data) to simultaneously estimate both the missing data model and the data analysis model. In the regression models, the dependent variables were dietary intake (nutrients and food groups), physical activity, sedentary behavior (inactivity and screen time), anthropometric measures and behavioral determinants at follow-up, with group (intervention or control group) as independent variable. Linear mixed effects models were used where anthropometric and behavioral (determinants) outcomes at T1 or T2 were predicted by fixed effects for group (control *vs.* intervention), baseline measurements at T0, sex and age as well as the random (nested) effect of school. Other covariates included in the model for the behavioral outcomes were ethnicity and (school) socio-economic status. Linear mixed effects models were also used where dietary intake (food diary) and physical activity (ActiGraph) were predicted by fixed effects for group (control *vs.* intervention), time (T0, T2: cross-sectional time points), the interaction between group and time, sex and age, as well as the random (nested) effect of school. An additional covariate included in the model for the dietary outcomes was total energy intake. For the physical activity outcomes other covariates that were included in the model were, BMI SD score, and percentage of weekdays the child had worn the ActiGraph. A two-sided p value of <0.05 was considered statistically significant. All statistical analyses were performed using the program SPSS (Version 17.0; SPSS Inc., Chicago, IL, USA and Version 20.0; IBM Corp., Armonk, NY, USA) and R (version 2.14.1; R Foundation for Statistical Computing, Vienna, Austria).

## 3. Results

### 3.1. Participants’ Characteristics

A summary of the characteristics of the participants is given in Table 2. At baseline the study population consisted of 615 children (23 schools) in the intervention group and 497 children (22 schools) in the control group. Approximately 1/7 originated from non-Western countries. At the start of the intervention the prevalence of overweightness or obesity was 20.5%. The hip waist ratio was significantly higher in the control group at T0 (*t*-test, *p* < 0.01). There were no other significant differences in baseline characteristics between the intervention and control group. Non-response analysis showed that children who dropped out from the intervention group from T0 to T2 differed significantly with regard to their age from children who participated fully in the evaluation study. Older children from the intervention school dropped out more often, however the difference in mean age was only two months.

**Table 2:** Characteristics of the study population at baseline (T0), first (T1a) and second follow-up (T2).

	Intervention	Control	Intervention	Control	Intervention	Control
	T0	T0	T1a	T1a	T2	T2
	(*n* = 615)	(*n* = 497)	(*n* = 568)	(*n* = 496)	(*n* = 367)	(*n* = 471)
Schools	23	22	22	21	17	21
	*n^1^ (%)*	*n (%)*	*n (%)*	*n (%)*	*n (%)*	*n (%)*
Female	320 (52.0)	255 (51.3)	296 (52.1)	255 (51.4)	200 (45.9)	236 (54.1)
Ethnicity						
Western	534 (87.1)	411 (84.7)	495 (88.1)	403 (84.8)	318 (90.3)	372 (84.4)
Non-Western	79 (12.8)	74 (15.3)	67 (11.9)	72 (15.2)	34 (9.7)	69 (15.6)
BMI category						
Underweight	21 (4.2)	14 (3.4)	27 (5.4)	17 (3.6)	13 (3.7)	18 (4.0)
Normal Weight	386 (76.7)	308 (74.4)	369 (73.9)	353 (75.6)	267 (76.1)	329 (73.1)
Overweight	73 (14.5)	78 (18.8)	79 (15.8)	80 (17.1)	58 (16.5)	85 (18.9)
Obese	23 (4.6)	14 (3.4)	24 (4.8)	17 (3.6)	13 (3.7)	18 (4.0)
	*mean ± SD*	*mean ± SD*	*mean ± SD*	*mean ± SD*	*mean ± SD*	*mean ± SD*
Age	9.2 ± 0.6	9.1 ± 0.6	9.7 ± 0.6	9.7 ± 0.7	11.2 ± 0.6	11.1 ± 0.6
BMI SD score	0.6 ± 1.2	0.6 ± 1.1	0.6 ± 1.2	0.5 ± 1.2	0.6 ± 1.1	0.6 ± 1.2
Hip/Waist ratio SD score	0.3 ± 1.3	0.5 ± 1.0 **	0.5 ± 1.8	0.3 ± 1.5	0.3 ± 0.9	0.4 ± 0.9

^1^ *n* varies due to missing data. ** *p* < 0.01.

### 3.2. Dietary Intake

The mean difference of one-day food consumption based on the food diary/24-h recall in the subsample showed no difference in energy intake and intake of macronutrients between intervention and control group. Similarly, the mean intake of fruit and beverages did not differ significantly between the intervention group and control group, however a trend effect was found on vegetable consumption (*p* < 0.10), due to an improvement in the control group that had a relatively low vegetable intake at baseline (Table 3).

Regarding dietary behavior measured by the questionnaire, no statistically significant results were found, except for consumption of sweet snacks (Table 4). A result was found from T0 to T1a (OR 1.60 (intervention *vs.* control); 95% CI 1.00; 2.55); the percentage of children in the intervention group that consumed sweet snacks daily was almost constant (T0 21.9%, T1a 22.3%) while the percentage in the control group decreased from 22.2% at T0 to 15.2% at T1a. Subgroup analysis showed that this effect was significant in boys and not in girls. At the later follow-up this effect disappeared.

**Table 3:** Mean values and estimated differences in dietary intake based on one-day food diary/24-h recall for children in the random subsamples at baseline (T0) and second follow-up (T2).

	Intervention	Control	Intervention	Control	Difference ^2,3^
	T0	T0	T2 ^1^	T2 ^1^	*B (95% CI)* ^5^
	(*n* = 98)	(n =100)	(*n* = 99)	(*n* = 120)	
	*Mean ^4^ ± SD*	*Mean ± SD*	*Mean ± SD*	*Mean ± SD*	
Total energy (kcal) ^t^	1815± 450	1850 ± 440	2024 ± 464	1991 ± 470	0.06 (−0.03 to 0.14)
Total protein (en%) ^t,6^	14.3 ± 3.8	14.2 ± 4.0	13.3 ± 2.9	13.5 ± 2.8	0.02 (−0.01 to 0.06)
Total fat (en%)	29.9 ± 6.0	30.6 ± 6.6	29.6 ± 6.1	30.6 ± 6.3	−0.54 (−2.9 to 1.78)
Total saturated fat (en%)	11.2 ± 2.8	12.0 ± 3.1	11.4 ± 2.9	11.9 ± 2.9	0.23 (−0.88 to 1.34)
Total carbohydrates (en%)	53.8 ± 7.2	53.1 ± 6.7	55.1 ± 6.4	53.8 ± 7.1	0.75 (−1.88 to 3.40)
Total mono- and disaccharides (en%)	30.4 ± 7.2	29.6 ± 5.9	30.9 ± 6.9	29.7 ± 7.7	0.34 (−2.31 to 2.99)
Fiber (g) ^t^	16.9 ± 6.3	17.0 ± 6.9	17.4 ± 4.9	17.9 ± 6.0	−0.04 (−0.15 to 0.07)
Breakfast					
Energy (en%) ^t^	16.1 ± 5.6	16.8 ± 6.1	15.9 ± 6.2	16.4 ± 6.8	0.03 (−0.13 to 0.18)
Amount of fruits (g) ^t^	105 ± 104	117 ± 94	122 ± 106	118 ± 107	−0.12 (−0.39 to 0.15)
Amount of vegetables (g) ^t^	96 ± 76	68 ± 68	85 ± 82	87 ± 85	−0.29 (−0.60 to 0.02) *
High fat or high energy snacks					
Energy (en%) ^t^	18.7 ± 11.3	17.4 ± 10.3	20.5 ± 10.6	17.9 ± 10.5	0.07 (−0.16 to 0.29)
Total beverages					
Energy (en%) ^t^	19.4 ± 7.0	19.2 ± 7.2	20.3 ± 7.2	19.0 ± 7.4	0.10 (−0.07 to 0.28)
Sugar sweetened beverages (en%) ^t^	12.1 ± 7.9	11.7 ± 7.6	12.9 ± 8.0	12.2 ± 7.6	0.04 (−0.18 to 0.26)
Fruit juices (en%) ^t^	1.6 ± 3.2	1.5 ± 3.0	2.3 ± 4.5	1.4 ± 2.6	0.24 (−0.05 to 0.55)
Other beverages (en%) ^t^	5.7 ± 5.5	6.1 ± 5.8	5.1 ± 4.6	5.4 ± 5.1	0.12 (−1.43 to 0.38)

^1^ Follow-up outcomes presented for the control group and intervention group are unadjusted; ^2^ Outcomes were predicted by fixed effects for group (control *vs.* intervention), time (T0, T2: cross-sectional time points), the interaction between group and time, sex, age, total energy intake; ^3^ Regression estimates were calculated accounting for clustering of observations within schools; ^4^ Means are from original variables; ^5^ CI = confidence interval; ^t^ Outcomes were log transformed; ^6^ en% = percentage of total energy (proportion of total energy intake from protein, fat, and carbohydrates); * *p* < 0.10.

### 3.3. Physical Activity

Mean daily physical activity level and time spent on moderate to vigorous activity, based on the ActiGraph, did not differ significantly between the intervention group and control group (Table 5). A trend effect (*p* < 0.10) could be observed for mean time spent inactive. Inactivity increased more in the control group than in the intervention group between T0 and T2.

No statistically significant result was found for the effect of the intervention on sedentary behavior (screen time) and physical activity behavior measured by the questionnaire. The majority of children in both the intervention and the control group seemed to be moderately active throughout the intervention period. On the other hand, the percentage of children that met the Dutch recommendation of being physically active for at least one hour a day increased by approximately 10% throughout the two years in both groups (Table 4).

### 3.4. Anthropometric Measurements

Overweightness prevalence (including obesity) increased from 22.2% to 22.9% in the control schools and from 19.1% to 20.2% in the intervention schools after two school years (Table 2). Results of the multi-level analyses of the BMI SD adjusted for baseline value, age and sex of the children did not show significant intervention effects. No significant intervention effects were found on hip and waist circumference (Table S3 in Supplementary Materials).

### 3.5. Behavioral Determinants

A statistically significant effect was found on knowledge between baseline and follow-up at the end of the first and second school year (T1a and T1b). The scores on knowledge increased in the intervention group as well as in the control group, however, the intervention group improved their score more (T0–T1a: β 0.29, CI 0.04; 0.55; T0–T1b: β 0.36, CI 0.06; 0.65) (Table 6). No significant intervention effects were found on the determinants of dietary, physical activity and sedentary behavior (screen time).

**Table 4:** Behavioral outcome measures according to the questionnaire responses at baseline (T0), first (T1a) and second follow-up (T2).

	Intervention	Control	Intervention	Control	T1 Effect of Intervention	Intervention	Control	T2 Effect of Intervention
	T0	T0	T1a ^1^	T1a ^1^	(T1a–T0) ^2,3^	T2 ^1^	T2 ^1^	(T2–T0) ^2,3^
	(*n* = 608) ^4^	(*n* = 469)	(*n* = 529)	(*n* = 418)	*B (95% CI)*	(*n* = 292)	(*n* = 404)	*B (95% CI)*
	*Mean ± SD*	*Mean ± SD*	*Mean ± SD*	*Mean ± SD*		*Mean ± SD*	*Mean ± SD*	
Sedentary behavior (screen time, range 1–3) ^5^	2.0 ± 0.7	2.0 ± 0.7	2.0 ± 0.7	1.9± 0.7	0.04 (–0.08–0.16)	2.2 ± 0.7	2.2 ± 0.6	0.02 (−0.10–0.15)
	*%*	*%*	*%*	*%*	*OR (95% CI)*	*%*	*%*	*OR (95% CI)^6^*
Norm active physical activity(≥ 60 minutes/day)	75.6	74.2	85.4	88.6	0.67 (0.40–1.13)	87.6	86.2	0.98 (0.82–1.7)
Dietary behavior								
Daily breakfast	89.0	84.6	89.2	88.8	0.71 (0.40–1.26)	89.8	89.8	0.91 (0.74–1.12)
Daily fruit	45.5	52.9	44.4	53.1	0.96 (0.67–1.38)	40.1	51.4	0.95 (0.84–1.09)
Daily vegetables	35.0	39.3	35.0	39.5	1.01 (0.69–1.47)	37.4	44.5	0.95 (0.83–1.09)
Daily soft drinks	41.9	34.9	42.7	32.4	1.14 (0.77–1.69)	36.0	28.3	1.03 (0.90–1.19)
Daily savory snacks	12.8	13.3	13.4	13.6	1.00 (0.59–1.72)	11.3	8.0	1.15 (0.94–1.41)
Daily sweet snacks	21.9	22.2	22.3	15.2	1.60 (1.00–2.55)	19.2	14.0	1.12 (0.95–1.32)

^1^ Follow-up outcomes presented for the control group and intervention group are unadjusted; ^2^ Outcomes were predicted by fixed effects for group (control *vs.* intervention), baseline measurements at T0, sex, ethnicity, age, socio economic status (SES); ^3^ Regression estimates were calculated accounting for clustering of observations within schools; ^4^ n varies due to missing data; ^5^ Mean of 1 active, 2 moderately active, and 3 inactive; ^6^ OR = Odds Ratio (intervention *vs.* control), CI = confidence interval.

**Table 5:** Estimated differences in physical activity outcomes based on ActiGraph data for children in the random subsample at baseline (T0) and second follow-up (T2).

	Intervention	Control	Intervention	Control	Difference ^2,3^
	T0	T0	T2 ^1^	T2 ^1^	*B (95% CI)* ^5^
	(*n* = 41)	(*n* = 37)	(*n* = 40)	(*n* = 52)	
	*Mean ^4^ ± SD*	*Mean ± SD*	*Mean ± SD*	*Mean ± SD*	
Inactivity (minutes)	512.9 ± 48.8	479.9 ± 64.9	528.9 ± 68.1	527.5 ± 64.0	−33.31 (−70.27 to 3.65) *
Moderate to vigorous physical activity ^t^ (minutes)	15.4 ± 9.0	21.4 ± 15.5	11.1 ± 7.2	14.0 ± 14.1	0.10 (−0.09 to 0.30)
Number of counts per minute ^t^	506.7 ± 122.8	566.9 ± 169.8	553.1 ± 135.1	563.7 ± 197.1	0.06 (−0.02 to 0.13)

^1^ Follow-up outcomes presented for the control group and intervention group are unadjusted; ^2^ Outcomes were predicted by fixed effects for group (control *vs.* intervention), time (T0, T2: cross-sectional time points), the interaction between group and time sex, age, BMI SD score, percentage ActiGraph worn on a weekday; ^3^ Regression estimates were calculated accounting for clustering of observations within schools; ^4^ Means are from original variables; ^5^ CI = confidence interval; ^t^ Outcomes were log transformed. * *p* < 0.10.

**Table 6:** Behavioral determinants (composite scores) according to the questionnaire responses at baseline (T0) and follow-up (T1a,T1b).

	Intervention	Control	Intervention	Control	T1a Effect of Intervention ^2,3^	Intervention	Control	T1b Effect of Intervention ^2,3^
	T0	T0	T1a ^1^	T1a ^1^	(T0–T1a)	T1b ^1^	T1b ^1^	(T0–T1b)
	(*n* = 604) ^4^	(*n* = 465)	(*n* = 526)	(*n* = 410)		(*n* = 292)	(*n* = 399)	
	*%* ^5^	*%*	*%*	*%*	*OR (95% CI) ^6^*	*%*	*%*	*OR (95% CI)*
**Determinants of behavior**								
Breakfast	62.7	60.3	64.3	61.9	1.01 (0.70–1.47)	71.2	65.8	1.05 (0.85–1.30)
Fruit intake	54.8	56.0	-	-	n.a ^7^	48.6	54.5	0.91 (0.74–1.12)
Vegetables intake	45.8	47.4	-	-	n.a.	50.0	52.5	0.95 (0.78–1.17)
Physical Activity	54.0	52.9	55.4	53.3	1.02 (0.71–1.46)	57.2	55.4	1.00 (0.82–1.23)
	*mean* *± SD ^8^*	*mean* *± SD*	*mean* *± SD*	*mean* *± SD*	*B (95% CI)*	*mean* *± SD*	*mean* *± SD*	*B (95% CI)*
Determinants of sedentary behavior (screen time, range 6–30)	20.2 ± 4.5	20.8 ± 4.2	20.3 ± 4.1	20.7 ± 4.1	0.37 (−0.37–1.11)	19.9 ± 4.0	20.7 ± 3.8	−0.04 (−0.86–0.78)
Knowledge score (range 0–9)	3.8 ±1.4	3.9 ± 1.5	4.2 ± 1.5	4.0 ± 1.5	0.29 (0.04–0.55) *	4.8 ± 1.4	4.5 ± 1.5	0.36 (0.06–0.65) *

^1^ Follow-up outcomes presented for the control group and intervention group are unadjusted; ^2^ Outcomes were predicted by fixed effects for group (control *vs.* intervention), baseline measurements at T0, sex, ethnicity, age, socio-economic status (SES); ^3^ Regression estimates were calculated accounting for clustering of observations within schools; ^4^ n varies due to missing data; ^5^ % High composite score of attitude, social norm, perceived behavioral control and intention; ^6^ OR = Odds Ratio (intervention *vs.* control), CI = Confidence Interval; ^7^ n.a. = not applicable; ^8^ Mean composite score of attitude, social norm, perceived behavioral control and intention. * *p* < 0.05.

## 4. Discussion

This study examined the effectiveness of the education program EF! in preventing and reducing overweightness of primary school children. Main results showed no effects of EF! on anthropometric measures or behavioral determinants. A positive effect on knowledge (measured by the questionnaire) and a positve marginal effect for mean time spent inactive (measured by the ActiGraph) for the intervention group were found. Furthermore, there were some effects measured on daily consumption of sweet snacks (questionnaire results) and vegetable consumption (one-day food diary results). In both cases, the behavior of the control group improved, however, baseline levels for vegetable consumption were lower in the control group compared to the intervention group.

The effect on knowledge was also found in other studies [31,32]. Two reviews showed that a majority of interventions targeting energy balance behavior were effective in changing knowledge, which was identified as a potential mediator of school-based lifestyle interventions [31,33]. Research showed also an effect of behavioral interventions on inactivity and sedentary behavior [14,15]. The effect on change of knowledge proves that the intervention has reached the children, however the next steps in behavior change such as change of attitude and intention could not be proven. An effect on the overall composite scales of behavioral determinants was absent. Additional analysis of separate scales on attitudes, perceived social norms, perceived behavioral control and intentions toward the dietary and physical activity behaviors similarly did not show an effect. A lack of change in other outcomes such as BMI by interventions with an educational component was also observed in similar studies [11,12,33]. In our study, this can be explained by the fact that the number of schools that completed the whole intervention program was limited. We performed a dose–response analysis of the relationship between the number of lessons given by the teachers and study outcomes in the intervention group, however an association was not found. The process evaluation showed that the children appreciated the program less in the second year. Additionally, fewer children perceived the lessons as difficult, which might indicate that it was less challenging and motivating for them. Besides, the involvement of parents appeared to be limited, while this is recognized as an important aspect for success [5,34,35].

The result of improvements in dietary intake in the control group *vs.* the intervention group was unexpected. We would have expected improvements (lower consumption of sweet snack and/or higher consumption of vegetables) in the intervention group, while the opposite was measured. An explanation for the effect on vegetable intake in the control group may be that there was room for improvement due to its lower baseline level. Another explanation may be an increase of the awareness caused by the intervention that may have led to a more critical assessment of dietary behaviors in the participants than in the control group. Regarding physical activity, both the intervention and control group showed a 10% increase of children that met the Dutch recommendation of being physically active for at least one hour a day. It is not clear whether the lack of difference can be explained by the content and intensity of the education on physical activity and nutrition. Process evaluation showed that in many control schools the same topics as represented in the EF! intervention were covered, although the time investment in control schools was lower than in the intervention school. The time investment of schools into health education therefore did not seem to be a distinctive factor, except for the improvement of knowledge. Another possible explanation is the schools’ decision to voluntarily participate in this study and the random assignment to the control group. This could have diminished the effects of the intervention, since the control schools could also have had the best intentions to prevent overweightness.

### 4.1. Strengths and Limitations of the Present Study

Strengths of the study included the randomized controlled design, strong theoretical foundation of the intervention based on the Intervention Mapping method, the duration of the program of two school years and the focus on targeting multiple behaviors related to a healthy lifestyle [5]. The randomization of schools resulted in an intervention and control group with fairly equal characteristics, although dropout rates were higher in the intervention group. Children of slightly higher age dropped out more often. We controlled for background characteristics in the analyses.

Estimation of behavior and behavioral determinants in a self-report questionnaire may have been difficult for children. Therefore, this type of assessment should be interpreted cautiously. To control for these shortcomings, food diaries, data from accelerometry and objective anthropometric measures were obtained in addition to the questionnaires. The findings of these measures were in line with the findings of the self-report questionnaire.

A limitation is the small study group sizes due to loss to follow up at T2 and limited availability of accelerometers. This may have affected the power to show significant statistical associations. For this reason, we mentioned the marginal significant effect on mean time spent inactive, measured by accelerometers. Another limitation appeared from the process-evaluation of the intervention. Teachers rated the lessons of EF! not entirely positive. Preparation and implementation time was considered too demanding. In some schools, children were disappointed that practical assignments could not be carried out, while other, more theoretical assignments, were perceived as boring in the case of repetition. This could have weakened the effectiveness of EF!. Selection of children and parents with a positive attitude towards a healthy lifestyle could also have weakened the effect of the EF! intervention. Although a randomly selected subsample was taken to measure dietary intake and physical activity, it can be assumed that in both the intervention and control group selection of motivated children and parents among the respondents could have biased the results of the measurements in a positive way which could have threatened the validity of the outcomes of the food diary/24-h recall and accelerometer measurements. A last limitation is that we did not adjust body weight for clothes worn. This might have influenced the calculation of BMI and overweightness prevalence rates, however because the weighing procedures in the intervention and control group were identical, the absence of an adjustment for the weight of clothing will not have affected our conclusions about the effect of EF! on the children’s BMI.

### 4.2. Implications for Practice and Directions for Future Research

In the absence of an effect of the EF! intervention on health behavior, recommendations for a different approach are given. In several reviews, the involvement of parents appears to be an important aspect for success, particularly in younger children [5,34,35]. It is therefore advised that more intensive multicomponent interventions at the level of the individual child, family, school, school environment and society are needed to have a long lasting effect on healthy lifestyle [6,11,31]. Such interventions, embedded in a whole school and broader community approach, may be successful in involving parents. The Healthy Eating and Physical Activity in Schools (HEPS) tool can be used to develop an integral health promoting school approach, including a checklist for assessing the quality of school interventions [36].

The implementation of EF! was incomplete in many schools. The limited time available for health education was a bottleneck for the schools to carry out the complete curriculum. We recommend giving attention to the completeness of the implementation of school programs on dietary and physical activity behavior through additional training of teachers, although the time investment should be carefully reviewed. We also advise to alternate theory lessons within the limited available time with practical lessons focusing on “fun”, such as physical activity lessons as children appreciated the “fun factor” in the program. The physical activity exercises and practical lessons on nutrition were valued most. It would be wise to invest in more innovative interventions to stimulate health behavior, compared to conventional methods [11,12]. Intervention developers need to search for strategies that fit more the wishes of the children, such as gaming or interactive, online education lessons.

The information transfer to the children in the intervention schools led to knowledge improvement during the two school years. Providing lessons in consecutive school years, with a continuing curriculum, is therefore recommended. Additional to the school curriculum, more attention should be paid to the school health policy.

Finally, it can be considered to integrate contact moments and activities outside school hours for children with overweightness or obesity in addition to a school multi-component strategy to achieve optimal results [37].

## 5. Conclusions

No major effects were found on BMI, waist and hip circumference, dietary intake and behavioral determinants. Evidence was found for increased knowledge and on the mean time spent inactive. To increase the effect in preventing and reducing overweightness of primary school children, it is advised to invest more in parental involvement, attractive lessons to enlarge the acceptability of the program and multi-component environmental strategies focusing on both sides of the energy balance.

## Supplementary Materials

Supplementary materials can be accessed at: http://www.mdpi.com/2072-6643/8/1/0012/s1. Table S1. Overview of the lessons. Table S2. Overview of questionnaire items. Table S3. Estimated mean differences in anthropometric measurements at baseline (T0) and second (T2) follow-up.
