# Supplementary material for: Effects of a Theory-Based Education Program to Prevent Overweightness in Primary School Children
Source: Nutrients. 2016 Jan 4;8(1):12. doi: 10.3390/nu8010012 (PMC4728626; doi:10.3390/nu8010012)
Supplement: Supplementary file 1 [file nutrients-08-00012-s001.docx]

Supplementary Materials: Effects of a Theory-Based Education Program to Prevent Overweight in Primary School Children

Paul L. Kocken, Anne-Marie Scholten, Ellen Westhoff, Brenda P.H. de Kok, Elisabeth M. Taal and R. Alexandra Goldbohm

**Table S1.** Overview of the lessons.

| **Grade** | **Lesson** | **Subject** | **Content** |
| --- | --- | --- | --- |
| 4 | 1 | Why exercising? | DVD |
|  |  |  | Physical activity: balloon jumping |
|  |  |  | Keep and discuss physical activity diary |
|  |  |  | Intensive heart rate and breathing exercises |
|  | 2 | Tasty from the Food Pyramid | List and discuss dietary habits |
|  |  |  | Assignments about the Food Pyramid (Dutch: Schijf van Vijf’) |
|  |  |  | Experiments with nutrients |
|  | 3 | Exercise and more exercise | Keep and discuss screen time diary |
|  |  |  | Physical activity: dance on music |
|  |  |  | Inventing games for inside and outside |
|  |  |  | Interview with parents or grandparents about games they used to play |
|  |  |  | Testing the invented games |
|  | 4 | Time for breakfast | List and discuss dietary habits |
|  |  |  | Assignments about own breakfast and inventing new breakfast recipes |
|  |  |  | Baking and tasting bread |
|  | 5 | Exercise, more exercise and keep exercising | Recognize sports and able to list favorite sports |
|  |  |  | Physical activity: pass through game |
|  |  |  | Interview with classmate about sport |
|  |  |  | Make report about sport |
|  | 6 | Fruit and vegetables | Fruit and vegetables test |
|  |  |  | Keep and discuss nutrition diary |
|  |  |  | Recognizing fruit and vegetables |
|  |  |  | Tasting fruit |
|  |  |  | Making fruit smoothies |
|  | 7 | Healthy choices | Develop healthy dietary behaviors |
|  |  |  | Physical activity: Commando (game) |
|  |  |  | Measuring energy expenditure |
|  |  |  | Evaluation of behavior after EF! lessons |

**Table S1.** *Cont.*

| **Grade** | **Lesson** | **Subject** | **Content** |
| --- | --- | --- | --- |
| 5 | 1 | Healthy = being fit! | Refresh knowledge from grade 4: question and answer game |
|  |  |  | Inventing physical activity games |
|  |  |  | Physical activity: pass and through game with music |
|  | 2 | Thick, thin, everybody is unique! | Interview with parents or grandparents about their childhood |
|  |  |  | Discussion about peer pressure, fashion and thick/thin |
|  |  |  | Assignment to look at own body and character |
|  |  |  | Exercise in being positive and giving compliments |
|  | 3 | Investigate your habits | Keep and discuss physical activity, sedentary and dietary scores, both individually and collective |
|  |  |  | Make nutrition diary of that day |
|  |  |  | Physical activity: follow the leader (game) |
|  | 4 | In balance? | List nutrition and physical activity behavior and energy values |
|  |  |  | Assignment about calories |
|  |  |  | Physical activity: Dice-exercise game |
|  |  |  | Calculate and discuss energy balance |
|  | 5 | Drinking and exercising | List and discuss drinking behavior |
|  |  |  | Interview about physical activity |
|  |  |  | Physical activity: Alphabet-game |
|  |  |  | Assignment about labels on drinks |
|  |  |  | Make a sport drink in class |
|  | 6 | Exercising and snacking | Snack detective: searching snacks at home and snacks from the Food Pyramid (Dutch: Schijf van Vijf) |
|  |  |  | Make a snack-counter |
|  |  |  | Bake popcorn |
|  |  |  | Make fruit and vegetable skewers |
|  | 7 | To school and back | Keep and discuss physical activity, sedentary and dietary scores, both individually and collective |
|  |  |  | Assignment about walking and cycling |
|  |  |  | Make a healthy lunch |
|  |  |  | Physical activity: photographer (game) |
|  | 8 | Eating and exercising at home | Interview at home about family recipe |
|  |  |  | Compare nutrition and physical activity behavior with friends |
|  |  |  | Inventing new physical activity games |
|  | 9 | We choose healthy! | Evaluation of behavior after EF! lessons |
|  |  |  | Self-test |
|  |  |  | Play healthy tag |

**Table S2.** Overview of questionnaire items.

| **Concept** | **Range/Answer Categories** | **Items** | **Example of Question** |
| --- | --- | --- | --- |
| Demographics | Does not apply | 8 | Are you a girl or a boy? |
| Physical activity behavior | Different ranges | 10 | How many days a week do you walk or bike to school yourself? |
|  |  |  | How long does it usually take to go from home to school? |
| Sedentary behavior (screen time) | 5 to 8 answer categories | 4 | How many days a week do you watch TV/video/DVD? |
|  |  |  | How long do you usually watch TV/video/DVD a day? |
| Nutrition behavior | 4 to 8 answer categories | 11 | How many days a week do you have breakfast? *This is the meal after getting up in the morning.* |
|  |  |  | How many days a week do you eat fruit? *(apples, oranges, tangerines, bananas, et cetera)* |
|  |  |  | On the days that you eat fruit, how many portions/servings do you eat usually a day? |
|  |  |  | *Examples of 1 portion/serving of fruit are: 1 apple, 1 banana, 1 peach, 1 kiwi, 2 tangerines, 2 plums, a handful of grapes, cherries or strawberries* |
| Knowledge | 3 to 5 categories | 7 | Breakfast is the most important meal of the day to be good at learning at school. |
| Cognitive attitude toward the frequency of behavior | 5-point Likert scale | 7 | Do you think that you eat enough vegetables? |
| Affective attitude | 5-point Likert scale | 14 | I think of having daily breakfast as good |
|  |  |  | I find a breakfast tasteful |
| Perceived social norm of parents | 5-point Likert scale | 7 | What do you think: Do your parents think that you should have breakfast every day? |
| Self-efficacy | 5-point Likert scale | 7 | How difficult do you find the following things? Having breakfast every day |
| Intention | 5-point Likert scale | 7 | Are you planning to have breakfast every day the next half year? |

**Table S3.** Estimated mean differences in anthropometric measurements at baseline (T0) and second (T2) follow-up.

|  | **Intervention** | **Control** | **Intervention** | **Control** | **Difference ^2,3^** |
| --- | --- | --- | --- | --- | --- |
|  | **T0** | **T0** | **T2 ^1^** | **T2 ^1^** | ***B (95% CI)* ^5^** |
|  | ***n* = 499 ^4^** | ***n* = 391** | ***n* = 348** | ***n* = 442** |  |
|  | ***Mean ± SD*** | ***Mean ± SD*** | ***Mean ± SD*** | ***Mean ± SD*** |  |
| BMI SD score | 0.56 ± 1.14 | 0.61 ± 1.12 | 0.55 ± 1.11 | 0.57 ± 1.17 | 0.06 (−0.16–0.28) |
| Waist SD score | 0.43 ± 1.02 | 0.44 ± 1.02 | 0.44 ± 1.01 | 0.43 ± 1.06 | 0.03 (−0.17–0.24) |
| Hip SD score | 0.09 ± 1.91 | 0.02 ± 1.10 | 0.24 ± 1.01 | 0.21 ± 1.07 | −0.03 (−0.29–0.24) |
| Hip/Waist ratio | 0.26 ± 1.27 | 0.46 ± 1.02 | 0.32 ± 0.90 | 0.38 ± 0.85 | 0.13 (−0.06–0.33) |

^1^ Follow-up outcomes presented for the control group and intervention group are unadjusted;
^2^ Outcomes were predicted by fixed effects for group (control *vs.* intervention), baseline measurements at T0, sex, age; ^3^ Regression estimates were calculated accounting for clustering of observations within schools; ^4^ *n* varies due to missing data; ^5^ CI = confidence interval.
